# Cannabis Intoxication Does Not Impact Nutritional Status in Patients With Small Burns

**DOI:** 10.1093/jbcr/irag027

**Published:** 2026-02-17

**Authors:** Sarah Wang, Eloise Stanton, Amara Emeh, Artur Manasyan, Sunnie Wong, Elizabeth Boudiab, Paige Baranco, Maxwell B Johnson, T Justin Gillenwater

**Affiliations:** Keck School of Medicine, University of Southern California, Los Angeles, CA 90033, United States; Division of Plastic and Reconstructive Surgery, Keck School of Medicine, Los Angeles, CA 90033, United States; Keck School of Medicine, University of Southern California, Los Angeles, CA 90033, United States; Keck School of Medicine, University of Southern California, Los Angeles, CA 90033, United States; Surgical Critical Care/Burn, Keck School of Medicine, Los Angeles, CA 90033, United States; Division of Plastic and Reconstructive Surgery, Keck School of Medicine, Los Angeles, CA 90033, United States; Division of Plastic and Reconstructive Surgery, Keck School of Medicine, Los Angeles, CA 90033, United States; Division of Plastic and Reconstructive Surgery, Keck School of Medicine, Los Angeles, CA 90033, United States; Surgical Critical Care/Burn, Keck School of Medicine, Los Angeles, CA 90033, United States; Division of Plastic and Reconstructive Surgery, Keck School of Medicine, Los Angeles, CA 90033, United States; Surgical Critical Care/Burn, Keck School of Medicine, Los Angeles, CA 90033, United States

**Keywords:** burn injuries, cannabis intoxication, nutritional status

## Abstract

Patients with burn injuries exhibit one of the most intense hypermetabolic responses among critically ill populations, making them highly susceptible to malnutrition—linked to prolonged hospital stays and delayed wound healing. While cannabis is recognized for its appetite-stimulating properties in acute settings, its association with the nutritional demands of burn injuries remains underexplored. A single-institution retrospective study was conducted on adult patients with burn injuries having < 20% TBSA who tested positive for cannabis on admission urine toxicology between 2015 and 2024. These patients were matched 1:1 with controls who tested negative for cannabis. The primary predictor variable was cannabis use, while outcomes included burn characteristics, prealbumin and albumin levels, overall outcomes, and complications. Significance was set at *P* < .05. We analyzed 76 cannabis-positive patients with burn injuries and 76 controls. No significant differences were found in demographics or outcomes. When controlling for body mass index, cannabis intoxication was not significantly associated with changes in admission prealbumin (18.8 vs 19.2, *P* = .804) or admission albumin (3.9 vs 4.0, *P* = .375) levels. There was also no significant variation in the number of days postadmission required to achieve peak prealbumin (3.8 vs 3.9, *P* = .876) and albumin level (0.3 vs 1.0, *P* = .088). Increased age was associated with a reduction in admission albumin (*P* < .001), and Caucasian patients had increased albumin compared to other races (*P* = .048). Cannabis intoxication had no significant association with preburn injury nutritional status. Further research with larger sample sizes is necessary to fully understand the complex relationship.

## INTRODUCTION

Cannabis use has become increasingly prevalent in the United States, with growing legalization for both medical and recreational purposes.^1^^,^^2^ The prevalence of cannabis use has been estimated to be up to 19.0% in 2021, with a projected increase over the coming years.^3^ The use of cannabis has been steadily increasing, likely in part due to changing state and federal policies, with many states legalizing it for recreational purposes, alongside growing public acceptance and expanding research into its therapeutic benefits.^1^

It is well known that cannabis-based products induce hyperphagia, increasing appetite and food cravings. Although not fully elucidated, its active compounds, particularly tetrahydrocannabinol (THC) and cannabidiol, interact with the endocannabinoid system to influence metabolic processes, energy balance, and immune responses.^4–7^ Cannabis influences appetite primarily through its activation of CB1 receptors in the hypothalamus.^7^ Moreover, THC, the psychoactive component of cannabis, enhances the release of ghrelin, a hormone that stimulates appetite, while also modulating dopamine pathways to increase the pleasure associated with eating.^8^

Patients with burn injuries experience one of the most extreme hypermetabolic responses in the critically ill population, placing them at high risk for malnutrition, which is associated with prolonged hospitalization and delayed wound healing.^9–11^ While cannabis is known to stimulate appetite in acute settings, its effects on the distinct metabolic challenges of burn injuries remain largely unexplored. In this study, we assess the effects of cannabis use on nutritional status in patients with acute burn injury. We propose that cannabis use may enhance preinjury nutritional status, potentially leading to improved clinical outcomes in patients with burn injuries.

## METHODS

This retrospective cohort study was conducted at a single American Burn Association–verified burn center and approved by the Institutional Review Board (#HS-23-00673). Given the retrospective design and minimal risk, a waiver of informed consent was granted.

### Study population

Adult patients with burn injuries, admitted between January 1, 2015 and December 31, 2024, were identified from the institutional burn registry. Inclusion criteria were: (1) a documented burn injury involving less than 20% TBSA, and (2) a urine toxicology screen performed at the time of admission. Patients were included in the cannabis-positive cohort if they tested positive for THC, the active compound in cannabis, on their admission toxicology screen. Cannabis-positive results included findings for “marijuana,” “cannabinoids,” or “THC.”

The control group was composed of adult patients with burn injuries who were admitted during the same time period but tested negative for cannabis on admission urine toxicology. Cannabis-positive patients were matched in a 1:1 ratio to cannabis-negative controls based on age (±5 years), gender, TBSA (±5%), and hospital length of stay (±2 days) using nearest-neighbor matching without replacement. Patients were excluded if they had incomplete laboratory data or lacked key outcome variables, including nutritional biomarkers.

### Data collection and variables

The primary predictor variable was cannabis intoxication status on admission. Primary outcome measures included serum prealbumin and albumin levels recorded on admission, as well as the time (in days postadmission) to reach peak prealbumin and albumin levels during the acute hospitalization period. Secondary outcomes included the presence of malnutrition at admission and discharge based on dietician notes (determined by nutrition-focused physical exam), C-reactive protein (CRP), the presence of inhalation injury, number of operative procedures, hospital length of stay, and mortality.

Demographic variables collected included age, sex, race, ethnicity, address, admission weight, and body mass index (BMI). Area Deprivation Index (ADI) scores were assigned to patients based on addresses and zip codes, and acted as a proxy for socioeconomic status. Higher scores indicated greater socioeconomic deprivation and resource scarcity. Patients were grouped into 3 categories based on ADI score: low (0-33), medium (34-66), and high (67-100). Clinical data were obtained from the electronic health record, including nutritional labs, operative notes, and discharge summaries. Nutritional biomarkers were analyzed using standard clinical laboratory protocols.

### Statistical analysis

The data were tested for normality using the Shapiro–Wilk test. Continuous variables were summarized using means and SDs, and categorical variables were reported as frequencies and percentages. Group comparisons were performed using Student’s *t*-tests for continuous variables and chi-square or Fisher’s exact tests for categorical variables. Multiple linear regression models were used to assess the association between cannabis use and nutritional biomarker levels, controlling for potential confounders including BMI, age, and race. Statistical significance was defined as a 2-tailed *P*-value less than .05. All analyses were conducted using Stata version 18.5 (Stata LLC) and R-studio.

## RESULTS

A total of 152 patients were included in the final analysis, comprising 76 cannabis-positive patients with burn injuries and 76 matched controls. There were no statistically significant differences in baseline demographic characteristics between the groups, including race (*P* = .494), ethnicity (*P* = .598), admission weight (*P* = .128), or alcohol use (*P* = .087) (Table 1). Clinical outcomes such as the number of complications (*P* = .495), number of operative procedures (*P* = .863), admission CRP (*P* = .436), and in-hospital mortality (*P* = .083) were also similar between groups (Table 2). However, cannabis-positive patients were significantly less likely to experience inhalation injury compared to controls (1.3% vs 6.6%, *P* = .023).

**Table 1:** Demographic and Burn Injury Characteristics of Adult Participants Who Were Cannabis Positive or Negative at the Time of Admission

	Cannabis positive (*n* = 76)	Cannabis negative (*n* = 76)	Overall (*n* = 152)	*P*-value
Age				
Mean (SD)	37.1 (17.0)	37.1 (16.9)	37.1 (16.9)	.996
Median [min, max]	30.4 [18.2, 85.7]	30.6 [14.3, 81.4]	30.6 [14.3, 85.7]	
BMI				
Mean (SD)	27.3 (6.9)	28.1 (7.6)	27.5 (7.1)	.162
Admission weight				
Mean (SD)	78.7 (20.6)	84.7 (27.2)	81.7 (24.3)	.128
Sex				
Female	20 (26.3%)	20 (26.3%)	40 (26.3%)	1.000
Male	56 (73.6%)	56 (73.6%)	112 (73.6%)	1.000
Race				
Asian	2 (2.6%)	4 (5.2%)	6 (3.9%)	.677
Black	15 (19.7%)	14 (18.4%)	29 (19.1%)	1.000
Native Hawaiian or Other Pacific Islander	1 (1.3%)	0 (0.0%)	1 (0.7%)	1.000
White	11 (14.5%)	17 (22.4%)	28 (18.4%)	.296
Other	47 (61.8%)	41 (53.9%)	88 (57.9%)	.411
Ethnicity				
Hispanic/Latino	39 (51.3%)	38 (50%)	77 (50.7%)	1.000
Not Hispanic/Latino	36 (47.3%)	35 (46.1%)	71 (46.7%)	1.000
Unknown	1 (1.3%)	3 (3.9%)	4 (2.6%)	.612
Comorbidities list				
Hypertension	7 (9.2%)	5 (6.6%)	12 (7.9%)	.764
Diabetes mellitus	8 (10.5%)	9 (11.8%)	17 (11.2%)	1.000
Obesity	6 (7.9%)	11 (14.5%)	15 (9.9%)	.303
Smoker	7 (9.2%)	4 (5.3%)	11 (7.2%)	1.000
Psychiatric illness	9 (11.8%)	9 (11.8%)	18 (11.8%)	1.000
Substance abuse	7 (9.2%)	11 (14.5%)	18 (11.8%)	.451
Alcohol use disorder	1 (1.3%)	5 (6.6%)	6 (3.9%)	.211
Wheelchair dependent	4 (5.3%)	1 (%)	5 (3.3%)	.363
Other	9 (11.8%)	12 (%)	21 (13.8%)	.638
Etiology of burns				
Chemical	2 (2.6%)	3 (3.9%)	5 (3.3%)	1.000
Cold	3 (3.9%)	4 (5.3%)	7 (4.6%)	1.000
Electrical	2 (2.6%)	8 (10.5%)	10 (6.6%)	.102
Friction	3 (3.9%)	2 (2.6%)	6 (3.9%)	1.000
Radiation	1 (1.3%)	1 (1.3%)	2 (1.3%)	1.000
Thermal	57 (75%)	47 (61.8%)	104 (68.4%)	.116
Unknown/Other	8 (10.5%)	11 (14.5%)	19 (12.5%)	.624
Inhalation^a^	1 (1.3%)	5 (6.6%)	5 (3.3%)	.023
Burn size (%)				
Mean (SD)	5.4 (3.9)	5.5 (4.1)	5.4 (4.0)	.877
Median [min, max]	5.0 [0.3, 16.5]	4.3 [0.4, 18.0]	4.5 [0.3, 18.0]	

Abbreviation: BMI, body mass index.

a It denotes significance.

**Table 2:** Clinical Outcomes of Adult Participants Who Were Cannabis Positive or Negative at the Time of Admission

	Cannabis positive (*n* = 76)	Cannabis negative (*n* = 76)	Overall (*n* = 152)	*P*-value
# Operative procedures				.863
1	20 (26.3%)	20 (26.3%)	40 (26.3%)	1.000
2	4 (5.3%)	7 (9.2%)	11 (7.2%)	.744
3	2 (2.6%)	1 (1.3%)	3 (1.3%)	1.000
Complications				
Mean (SD)	1 (1.64)	2.5 (1.73)	2.2 (1.64)	.495
Median [min, max]	1 [1, 1]	2 [1, 5]	2.0 [1, 5]	
Length of hospital stay				
Mean (SD)	9.6 (8.5)	8.9 (6.8)	9.2 (7.7)	.608
Median [min, max]	7.0 [2.0, 50.0]	7.0 [2.0, 35.0]	7.0 [2.0, 50.0]	
Mortality	0 (0.0%)	3 (3.9%)	3 (2.0%)	.083

Of those who had malnutrition assessments recorded in dietician notes, 22.6% (*n* = 14/62) of cannabis-positive patients and 24.4% (*n* = 10/41) of cannabis-negative patients were diagnosed with malnutrition at admission. There was no significant difference (*P* = .832) found between these cohorts in admission malnutrition. For cannabis-positive patients, 7 out of 14 (50%) maintained a diagnosis of malnutrition at discharge, and 6 out of 10 (60%) maintained this diagnosis in cannabis-negative patients.

After grouping patients into ADI categories, there were 13 (8.6%) patients with low ADI, 85 (55.9%) with medium, and 53 (34.9%) with high. No significant differences in ADI groupings were found between cannabis and control groups (*P* = .879).

It was found that admission prealbumin was normally distributed (*W* = 0.986, *P* = .140) and admission albumin was not normally distributed (*W* = 0.956, *P* < .001). There were no significant differences in nutritional markers at the time of admission (Table 3). Mean admission prealbumin levels were 18.8 mg/dL in cannabis-positive patients compared to 19.2 mg/dL in controls (*P* = .804), and mean admission albumin levels were 3.9 and 4.0 g/dL, respectively (*P* = .375). The average number of days postadmission required to reach peak prealbumin and albumin levels was also similar between groups (3.8 vs 3.9 days for prealbumin, *P* = .876; 0.3 vs 1.0 days for albumin, *P* = .088). In multivariable analysis adjusting for BMI, which was not significant, cannabis use was not independently associated with any nutritional biomarker. Notably, increased age was significantly associated with lower admission albumin levels (*P* < .001), and Caucasian patients had higher albumin levels compared to patients of other races (*P* = .048). It was also found that patients in the medium ADI cohort had lower admission prealbumin than the low ADI cohort (*P* = .010). However, there was no difference in admission prealbumin between the high ADI and low ADI cohorts (*P* = .112). There were no significant differences between ADI groups in admission albumin.

**Table 3:** Nutritional Markers in Cannabis vs Noncannabis Users

Variable	Cannabis positive	Cannabis negative	*P*-value
Admission prealbumin (SD)	19.0 (6.7)	19.2 (6.4)	.42
Admission albumin (SD)	4.0 (0.6)	4.0 (0.6)	.31
Δ Prealbumin	18.8 (6.3)	19.2 (6.3)	.804
Δ Albumin	3.9 (0.5)	4.0 (0.5)	.375
Days to peak prealbumin	3.8 (5.6)	3.9 (5.6)	.876
Days to peak albumin	0.3 (2.5)	1.0 (3.3)	.088
Admission CRP	67.9 (79.4)	78.9 (77.9)	.436
Discharge CRP	39.8 (48.2)	53.3 (60.2)	.240
Admission malnutrition	14 (18.4%)	10 (13.2%)	.832
Discharge malnutrition	7 (9.2%)	6 (7.9%)	.772

Abbreviation: CRP, C-reactive protein

## DISCUSSION

Patients with burn injuries are highly susceptible to malnutrition given the intense metabolic demands of the body to promote healing.^11^ During hospitalization, common practice to assess nutritional status includes measuring trends of serum proteins such as albumin and prealbumin. A study by Clark et al. found that in 2007, 86% of burn centers were using prealbumin and 46% of centers were using albumin as nutritional markers.^12^ With the rapidly rising incidence of cannabis use among patients with burn injuries and its known appetite stimulating properties, we hypothesized that cannabis use would be associated with improved nutritional status in acutely hospitalized patients with burn injuries. However, we found no significant differences in admission prealbumin or albumin levels, or in the time required to reach peak levels during hospitalization, between cannabis-positive patients and controls.

Cannabis is widely known to possess appetite-stimulating properties through activating hypothalamic areas involved in reward behaviors and feeding regulation.^7^^,^^13^ While cannabis doubles food ingestion, it has been shown that most of the energy gained is from carbohydrate and snack food intake.^14^ Patients with burn injuries have increased energy expenditures and more severe catabolism, especially of muscle protein.^11^ There is evidence that protein replacement postburn is beneficial to decrease muscle wasting.^11^ Given the research that cannabis increases carbohydrate and not protein intake, the profound hypermetabolic needs of burn injuries may be overwhelming any potential nutritional benefit from increased appetite. The increased energy expenditure required for wound healing can rapidly deplete nutritional stores, regardless of appetite.^11^

Furthermore, standard nutritional markers like albumin and prealbumin are good prognostic markers for morbidity and mortality, but may be worse nutritional markers since serum levels are acutely influenced by other inflammatory markers that are active during the postburn period.^12^^,^^15^^,^^16^ The finding of increased age being significantly associated with lower admission albumin is consistent with other studies showing that decreases in albumin correlates well with increased mortality.^15^ Caucasian patients having higher albumin levels than patients of other races supports the notion that other demographic and physiological factors play a significant role in determining these nutritional markers, potentially more so than cannabis use in this acute setting. Socioeconomic factors may also influence preinjury nutritional status, as patients in the medium ADI cohort had significantly lower admission prealbumin levels than the low ADI cohort. This suggests that baseline socioeconomic environment can be an underlying determinant of preinjury nutritional status. However, no difference was found between the high and low ADI cohorts, and overall ADI grouping was not significantly different between cannabis and control groups. This relationship between socioeconomic status and preinjury nutrition must be further studied to understand the intricacies that underlie these differences.

Beyond nutritional status, there were no statistically significant differences between cannabis-positive patients and controls in other acute clinical outcomes, including number of operative procedures, complications, or mortality. This suggests that cannabis positivity on admission does not appear to increase risk of these major adverse outcomes in an acute setting. This is consistent with other studies around substance use and outcomes of patients with burn injuries. While polysubstance use prolongs hospital stay, increases local wound infections, and is associated with more graft failure, it was always found upon adjusted analysis that cannabis itself did not worsen these outcomes.^17–19^ This highlights the importance of distinguishing cannabis use alone from polysubstance use when examining burn recovery trajectories.

While this study focuses on the acute hospitalization phase, cannabis may hold potential therapeutic relevance in the long-term phases of burn recovery. Burn survivors frequently experience chronic pain and itching, symptoms which are oftentimes managed in other dermatologic and systemic conditions with medical cannabis use.^20–22^ Furthermore, cannabis plays a significant role in reducing inflammation in keratinocytes in the skin, which raises the question of whether cannabis could influence postacute processes relevant to scar formation and pruritus.^23^ These potential therapeutic uses of cannabis in burn recovery are largely unexplored and unvalidated in the burn population.

Limitations of this study include being a single institution study with a smaller sample size, which limits the generalizability to the population of patients with burn injuries. Urine toxicology screening at admission provides a picture of recent cannabis exposure but is limited in revealing the extent and duration of cannabis use, and thus does not represent any associations with long-term cannabis exposure. There was also no differentiation between the types of cannabis used, and if it was recreational or medical. Finally, the reliance on standard clinical nutrition markers of albumin and prealbumin may not fully capture the complex nutritional status of patients with burn injuries in the acute state or all the impacts of cannabis on nutrient intake, as these markers are imperfect indicators of true nutritional status in the setting of acute inflammation. The admission values were used as the only consistently available objective data to infer a patient’s preinjury nutritional status; however, it is recognized that the change in these markers during the course of hospitalization is more reflective of the nutritional support provided than the preadmission cannabis exposure. Other nutritional markers such as metabolic cart results and micronutrient levels were searched for and not found in patient records.

Despite these limitations, this study sheds light on the underexplored relationship between cannabis use and acute nutritional status. Future studies are needed to capture more detailed information on cannabis use patterns, including frequency, duration, and type, to gather more information on cannabis as an appetite stimulant during admission. Future research should also use different comprehensive nutritional assessments to better understand the potential impact. The observed differences in nutritional markers related to socioeconomic status necessitate further exploration into potential mediators of this relationship, such as food insecurity and lack of resources. Lastly, there is still a dearth of research surrounding the influence of cannabis use on different burn outcomes such as pain management, psychological recovery, and long-term functional outcomes.

## CONCLUSION

This study was the first to investigate the association between cannabis positivity on admission and nutritional status in adult burn survivors with minor burn injuries. It was found that cannabis intoxication was not significantly associated with changes in albumin or prealbumin throughout acute hospitalization. Furthermore, cannabis use was not significantly linked with an increased risk of acute complications, length of stay, or mortality. The findings suggest that while cannabis use is increasingly prevalent in the population of patient with burn injuries, it does not appear to be linked with any nutritional benefit during burn injury recovery. In the clinical setting, standard nutritional assessments and interventions remain essential for all patients with burn injuries, and cannabis-using patients may not require different resource utilization than those that do not use cannabis.

## Notes

Author contributions: Sarah Wang (Conceptualization [lead], Data curation [equal], Methodology, Project administration, Writing—original draft [lead]), Eloise Stanton (Data curation [equal], Formal analysis, Investigation [lead], Writing—original draft [supporting]), Amara Emeh (Conceptualization [supporting], Data curation [equal], Writing—original draft [supporting]), Artur Manasyan (Data curation, Writing—original draft [supporting]), Sunnie Wong (Conceptualization, Project administration [supporting], Supervision [equal]), Elizabeth Boudiab (Conceptualization [supporting], Supervision [equal]), Paige Baranco (Methodology, Writing—review & editing [supporting]), Maxwell Johnson (Supervision, Writing—review & editing [equal]), and Justin Gillenwater (Conceptualization [equal], Supervision, Visualization, Writing—review & editing [lead])

Funding: *None.*
